# Medical Heresis, &c.

**Published:** 1881-02

**Authors:** Walter M. James


### BOOK NOTICES AND REVIEWS.

MEDICAL HERESIES: HISTORICALLY CONSIDERED. By Gonzalvo C. Smythe, M.D., Phila., Presley Blakiston, 1880.

This little book gives a review of all the strange theories and singular practices that have prevailed in medicine from the earliest ages down to our own day, with a particular reference to homœopathy. It is indeed specially leveled at the latter, the other heresies being considered only by way of introduction.

Ever since Hahnemann first gave to the world his immortal discoveries that have led to a grand reform in the most important of all sciences—the science of medicine,—efforts more or less malignant have been made to excite contempt for his doctrines and break up the influence they have been exerting upon medical practice.

Most of these efforts have been conducted with a combination of malice, prejudice and partizanship truly astonishing. But as these passions were so plainly visible in the attacks, they have been comparatively unsuccessful; their influence having, if anything, the quality of increasing the number of the adherents of the hated system from the fact that unjust persecution stimulates investigation. In the little book before us there is a singular absence of these blinding passions in its treatment of the subject. Homœopathy is considered with a coolness, calmness and liberality that excites our surprise. This spirit of fairness will cause it to be extensively read, and to command a large share of respect. It must prove a very damaging blow to our school from which it will take us a long time to recover. When an allopathic doctor finds his patient going over to the new heresy he will simply place in his hands a copy of this book and thereby change his intention.

Nevertheless the author has not escaped committing one or two gross acts of injustice. Thus on pp. 149 to 153 he estimates the number of strokes necessary to make a given high potency and shows that the number is practically infinite. His method of doing this is peculiar. On page 150 he says: "Take, for instance, one drop of this mother tincture; this is mixed with ninety-nine drops of alcohol contained in a vial and having received twelve strokes is called the first dilution. The second dilution will consist of one hundred vials and twelve hundred strokes." Thus our worthy author makes out that having raised one drop of the first dilution to the second dilution, that we then proceed in the same way with each one of the remaining ninety-nine drops of that first dilution: that this same thing is continued with the second, and then with the third and so on. By magnifying in this untruthful way he calculates that the tenth dilution will require one quintillion vials and twelve quintillion strokes: whereas every homœopathist knows that when he asks for the tenth dilution at the pharmacy the preparation he gets has been through ten successive vials and has received one hundred and twenty strokes. Now, inasmuch as the author has already acknowledged that having taken a drop from any given dilution in order to make the next higher he throws away the remaining ninety-nine drops (or ninety-nine per cent. as he expresses it) the wilful injustice of these figures is perfectly apparent. By the same method the thirtieth potency would require one hundred and twenty sextillions of duodecillions of strokes (that is to say one million raised to its tenth power and the product multiplied by one hundred and twenty.) To accomplish this task would take more than six hundred and sixty-one quadrillions of decillions of years!* But the arithmetician has

* One million raised to the eighth power and the product multiplied by six hundred and sixty-one!

succeeded in deceiving himself, for he triumphantly exclaims (p. 153); "In the face of this calculation the principal homœopathic pharmaceutical establishments in the United States advertise that they have carried up to the two hundredth potency, two hundred and fifty remedies * * * can it be that the fool-killer has visited this planet since Hahnemann proposed this theory?" Evidently not, else the professor from Central College would not be alive to tell the above tale.

Again at pages 141 and 142 we get a very incomplete statement of the pathogenesis of Calc-carb. No mention is made of the great characteristics of this remedy by which we are enabled to make those wondrous cures which compel a large following of the despised creed. Why does he omit the perspiration on the head while sleeping, so profuse as to "wet the pillow far around;" the coldness, flabbiness, and paleness of the skin; the *disgust* for fat, butter, etc.?

The writer of this article was once undergoing an operation upon his teeth by a well-known dentist in this city when the dentist's little daughter three years old ran into the office. The dentist, who had no faith in homœopathy, spoke of a flow of bland pus from the child's right ear which had continued for six months and had resisted all treatment. The father jestingly asked us to cure the flow. We took the request seriously and immediately proceeded to get the "totality of the symptoms.' We were informed that the child perspired much about the head whilst sleeping. That the perspiration was apt to be sour, and that there were clay-colored passages from the bowels. The skin was cool, flabby and pale. Here was a group of symptoms calling for *Calc-Carb.*, which we were permitted to give the little patient. In one week the flow from the ear stopped and the perspiration ceased. When prescribing we had not recollected to ask whether the child disliked meat, but the parents voluntarily informed us that the infant had asked for both butter and meat neither of which could she previously be induced to touch. Now this desirable result was brought about by attention to the silly symptoms which a diseased fancy (?) and gross superstition (?) had developed as the toxic influence of a piece of inert oyster shell. Will our allopathic friend credit this statement, or will he mentally decide that we must be "either foolish or knavish"?

Chapters twelve to fourteen are devoted to a statement of Hahnemann's doctrines with a discussion of their merits. With but a few exceptions (two of which are already noted) its statements are true from an allopathic stand-point. In chapters fifteen and sixteen the fallacy of this doctrine is shown by the strongest of all evidence—the testimony of the men who are supposed to be its defenders and professors; thus getting a kind of states evidence. By quoting the assertions of a certain kind of pretenders to homœopathy he secures a mass of testimony that, if it is to be believed, renders his argument almost unanswerable.

But who are these men whose testimony is taken to refute the very doctrine they are supposed to uphold? We will answer. They are men who do not know, and never did know, any thing about the homœopathic principles; who never gave a homœopathic prescription; who, whilst professing the name, are really making war upon the cause; who are denying and denouncing cures made by infinitesemal means whilst continuing to sail under the flag for the sake of the trade advantage it gives them; who wish the favor of the public at the same time that they are striving to win the recognition of the old school that they may enjoy the trades-union advantages of the latter; who cater to the medical prejudices of the old school in every possible way; who denounce a consistent homœopathist with all the venom of an allopathist; get up clap-trap tests in which to ensnare the unwary, and seizing upon the unlucky deliverances of the late Dr. Dunham, clamor for "liberty of medical opinion and action;" who hold professorships in homœopathic colleges and brazenly declare that they give emetics, purgatives, etc., and consider themselves *very good* homœopathists; who talk learnedly of the "collateral sciences" and proclaim what no old school practitioner will venture to assert, that pathology is an "exact science."

One of them, devoting himself to the praiseworthy task of exposing the fallacy of prescribing inert substances, points the scornful finger at the adamantine rock, bids us consider the ways of nature, and then dramatically demands if *now* we believe Silicea soluble? Thus at one fell swoop he annihilates the whole Hahnemannian horde and even brings confusion upon the learned Storer's assertions in his "Dictionary of Solubilities" (art., Silicic Acid). Another warrior excitedly denies that he "sees one particle of proof that the dynamized remedy given singly and in the smallest dose * * * is alone homœopathic;" declares his opinion that "the man who never uses topical applications or mechanical appliances in non-surgical cases is unfaithful to his patients," and finally exclaims, "if this is heresy, make the most of it!"* Well, yes! that's precisely what Drs. Gonzalvo C. Smythe and H. C. Wood are doing this moment! We hope he is pleased with their work.

Do these champions of "liberty of medical opinion and action" gain the ardent desire of their hearts—the recognition and favor of the old school? Not at all. Dr. H. C. Wood the learned editor of the *Phila. Medical Times*, looking on with contemptuous smile penetrates their motives and derisively exclaims:

"Little by little is creeping out that which the regular profession has long known, namely, that for a man to be a homœopathic physician *at present*† necessitates that he be ignorant, foolish or knavish—that is, if it be knavish to live a lie."‡

And the author of the book under review assuming these persons to be "very good homœopathtc authority," makes the very strongest argument against homœopathy by quoting their writings. Thus on page 209 we find the following: "These propositions and conclusions of Dr. Dake, if accepted, settle the entire

---

* *Homœopathic Times*, Dec. 1880, p. 205.

† Italics ours. ‡ *Phila. Medical Times*, April 13th, 1878, p. 336.

question of homœopathy, and *concede almost every point which I have attempted to establish; not in regard to similia similibus curantur only, but also triturations, dilutions and dynamizations.*"*

Here is a munificent and desirable reward for their efforts to unite a counterfeit homœopathic practice with the old school. One noted authority of the latter accusing them of "living a lie," the other one quoting them only to annihilate them!

To cap the climax the author indulges a cheerful prediction:

Page 197: "A process of evolution has been going on and will continue until the most objectionable principles of the school will be eliminated." Darwin teaches that the process of evolution is attended with a "survival of the fittest." According to "Medical Heresies," the plain inference is that the fittest are those men who, Dr. Wood stoutly asserts, are "living a lie"!!

How do they like the prospect? Will they continue to bear this ignominious burden piled upon them by the scornful enemies whom they seem so anxious to placate? Are they so completely dominated by these two deceitful watchwords, "liberty of medical opinion and action," and "medical union," that they will patiently stand while the load is raised ever higher and higher, and these two absurd ideas lead them by the nose, as asses are, deeper and deeper into the slough of despond? But will the ultra Hahnemannians consent to share these desperate fortunes? Will they not rather disavow all fraternity with such people; combine to expel them from the ranks, and force them to assume the title that clearly describes their practice, namely: eclectic? Will they not, then, proceed to "eliminate" these eclectics, immediately, without waiting for the "process of evolution"? And when the separation is accomplished, will it not be possible to judge from the size of the respective fragments whether the Hahnemannians are a "split" or a "splinter"?*

This "heart of oak" is decayed. The "shins of the conceited

---

* *Hahnemannian Monthly*, Dec. 1880, p. 757.

young woodman" are in no danger; a blow from his axe can not "glance" for there is no elastic body of timber to resist it. On the contrary, it will only bury itself in a mass of rotten granulations. Let the woodman join the workman from Indiana, burst the outer shell, and turn the old log up to the light, and it will fall apart of its own rottenness.

Enough of this. The allopaths set themselves to ridiculing our principles, and proving their absurdity by copious quotations from the anti-Hahnemannians; yet all this is foreign to the question to be settled. The sole issue between homœopathy and allopathy is this: which is the better curative method *i. e.* which cures quickest, easiest, the most certainly, and leaves the least bad sequelæ? If this question be answered in favor of allopathy, then they need trouble themselves no further with unsuccessful homœopathy, for it will soon die a natural death. They need make no murderous assaults; they need waste no time scanning our literature for offensive weapons. On the other hand, if this question be fairly met and answered in favor of homœopathy *then* and *only then*, are the means by which this superior success is achieved to be discussed. If we use their methods then we do better with them than they do. If we employ other and newer methods they should expose the deception, if any there be, or failing to do so, adopt them. If they can find no deception and yet refuse to adopt them (always supposing that they are shown to be more successful *then* allopathy must stand self-accused of wilful neglect of life—aye, of wilful murder! Of all the many writers against homœopathy, none have attempted to prove that we are *less* successful in healing than they. They are too casuistic. They carefully avoid this point, preferring to appeal to vulgar prejudice, always existing against new and strange methods. The wonderful growth of homœopathy in spite of foes without and traitors. within, in spite of the extreme novelty of its tenets, is certainly a strong, *a priori* argument in favor of its being the better curative method.—WALTER M. JAMES.

